# Childhood Vaccination and Its Association With Bacterial Meningitis Incidence: Insights From a Pakistani Tertiary Center

**DOI:** 10.7759/cureus.95829

**Published:** 2025-10-31

**Authors:** Kiramat Ullah, Inam Ullah, Abbas Ali Khan, Amin Ullah

**Affiliations:** 1 Pediatric Medicine, Bacha Khan Medical College, Mardan Medical Complex, Mardan, PAK; 2 Pediatric Unit, District Headquarters (DHQ) Teaching Hospital, Mardan, PAK; 3 Pediatric Medicine, Medical Teaching Institution, Mardan Medical Complex, Mardan, PAK; 4 Pediatric Unit, Sajida Islam General Hospital Takht Bhai, Mardan, PAK

**Keywords:** bacterial meningitis, children, pakistan, streptococcus pneumoniae, vaccination

## Abstract

Background: Bacterial meningitis is a life-threatening infection in children under five years of age, most commonly caused by *Streptococcus pneumoniae*, *Haemophilus influenzae* type b (Hib), and *Neisseria meningitidis*.

Objective: To ascertain the frequency of bacterial meningitis in children who were fully or partially vaccinated according to the national Expanded Program on Immunization (EPI) schedule versus those unvaccinated. Secondary objectives included describing pathogen distribution and demographic characteristics in vaccinated versus unvaccinated children.

Methodology: A hospital-based cross-sectional study was conducted at the Department of Pediatrics, Mardan Medical Complex, from January to December 2023. Children aged two months to five years with suspected meningitis were enrolled using consecutive sampling. Vaccination status was verified through immunization cards; children with unverifiable vaccination histories were excluded. Children were categorized as fully vaccinated, partially vaccinated (missed some vaccines), or unvaccinated. Data were collected using a structured proforma and analyzed in SPSS version 26 (IBM Corp., Armonk, NY). Odds ratios (ORs) with 95% confidence intervals (CIs) were calculated to assess the association between vaccination and bacterial meningitis. Baseline differences in age and gender were tested statistically (t-test/chi-square), and Fisher’s exact test assessed differences in pathogen distribution.

Results: Of 280 enrolled children, 168 (60.0%) were vaccinated (fully or partially) and 112 (40.0%) were unvaccinated. Bacterial meningitis was confirmed in 60 cases (21.4%) by cerebrospinal fluid analysis, with a significantly lower frequency among vaccinated children (24/168; 14.3%) compared to unvaccinated children (36/112; 32.1%). Unvaccinated children were nearly three times more likely to develop meningitis (OR = 2.85, 95% CI: 1.56-5.21; p = 0.0006). *Streptococcus pneumoniae* was the most common pathogen (40.0%), followed by Hib (21.7%) and *Neisseria meningitidis* (13.3%); Gram-negative organisms were less frequent. Pathogen distribution did not differ significantly between vaccinated and unvaccinated groups (Fisher’s exact test, p = 0.41). Serotype-specific analysis was not performed.

Conclusion: Childhood vaccination according to the national schedule significantly reduces the risk of bacterial meningitis, with unvaccinated children nearly three times more vulnerable. Strengthening vaccine coverage, ensuring timely completion of immunization schedules, and addressing barriers, such as limited access, vaccine hesitancy, and administration by undertrained personnel (e.g., teachers, clerks, or newly recruited staff without proper knowledge or training), are essential to reduce disease burden.

## Introduction

Bacterial meningitis is one of the most serious and potentially fatal illnesses affecting children, characterized by inflammation of the meninges and associated with high morbidity and mortality worldwide [1,2]. Despite advancements in supportive care and antimicrobial therapy, outcomes remain suboptimal in many settings, particularly in resource-limited regions [3]. Survivors often experience long-term neurological sequelae, including hearing loss, cognitive impairment, seizures, and motor deficits, all of which contribute substantially to the public health burden [4].

The epidemiology of pediatric bacterial meningitis has shifted significantly in recent decades, largely due to widespread immunization programs [5]. Vaccines targeting *Streptococcus pneumoniae*, *Neisseria meningitidis*, and *Haemophilus influenzae* type b (Hib) have markedly reduced disease incidence globally, especially in countries with high vaccination coverage. However, regional disparities persist, reflecting differences in vaccine availability, uptake, and healthcare infrastructure [6]. In many low- and middle-income countries, vaccine hesitancy, insufficient funding, and structural deficiencies in healthcare delivery contribute to suboptimal immunization coverage, leaving children vulnerable to preventable infections [7].

In Pakistan, bacterial meningitis continues to be a significant public health concern, affecting thousands of children annually [8,9]. Although the introduction of Hib and pneumococcal conjugate vaccines into the Expanded Program on Immunization (EPI) has reduced case numbers, uneven coverage, limited public knowledge, and challenges in vaccine delivery prevent optimal protection [10]. Notably, in many areas, vaccines are often administered by non-health professionals, such as teachers, clerks, or newly recruited staff, who may lack proper training, leading to mishandling and reduced vaccine effectiveness. Children who remain unvaccinated are at higher risk of infection, severe complications, and mortality compared with their vaccinated peers [11].

In this study, the term “frequency” refers to the prevalence of confirmed bacterial meningitis cases among vaccinated and unvaccinated children presenting to a tertiary care hospital within the defined study period, rather than the community incidence. Understanding the prevalence in both vaccinated and unvaccinated populations is critical for identifying gaps in vaccination coverage, assessing vaccine effectiveness, and informing strategies to reduce disease burden. Hospital-based research in resource-limited settings, where national surveillance systems may be lacking, provides valuable insights into these issues. The research objective of this study was to ascertain the frequency of bacterial meningitis in children who are vaccinated versus those who are unvaccinated at the time of presentation to a tertiary care facility.

## Materials and methods

Study design and setting

This was a single-center, cross-sectional design study conducted at the Department of Pediatrics, Mardan Medical Complex, Mardan, over a period of one year. A cross-sectional design was chosen because it allowed estimation of the prevalence of bacterial meningitis among vaccinated and unvaccinated children at a single point of hospital presentation. While case-control or cohort designs could provide stronger causal inference, the cross-sectional approach was more feasible in terms of time, resources, and patient flow in a tertiary care setting.

Inclusion and exclusion criteria

The study included children of both sexes who presented with suspected meningitis and were aged between two months and five years. This age range was selected because bacterial meningitis is most common and clinically severe in this vulnerable group, and routine Hib and pneumococcal vaccinations are administered during this period. Newborns (neonates, defined as <28 days of age) were excluded to avoid confounding from organisms more typical of early-onset neonatal sepsis, such as group B *Streptococcus* and *Listeria monocytogenes*.

Children were excluded if they had incomplete or unverifiable vaccination history, defined as the absence of both an EPI card and reliable parental recall. Additionally, children vaccinated outside Pakistan with non-standard immunization records and those whose parents or guardians refused to provide informed consent were not included. The exclusion of unverifiable vaccination records was necessary to ensure internal validity, although it may have reduced external validity. A sensitivity analysis was considered but not performed due to the small number of unverifiable cases.

Sample size

A total of 280 children were enrolled using consecutive sampling from the inpatient and outpatient pediatric population at Mardan Medical Complex. Consecutive sampling was chosen to minimize selection bias and capture all eligible cases during the study period. We acknowledge that consecutive sampling could be influenced by seasonal variation in meningitis incidence, which may bias results. A formal a priori power calculation was performed and indicated that the study had >80% power to detect the observed difference in prevalence of bacterial meningitis between vaccinated and unvaccinated groups at α = 0.05 using $n = \frac{\left( Z_{\alpha/2} \sqrt{2P(1-P)} + Z_{\beta} \sqrt{P_1(1-P_1) + P_2(1-P_2)} \right)^2}{(P_1 - P_2)^2}$. The final sample size is broadly comparable to other observational studies on pediatric meningitis frequency [12].

Data collection

Demographic data, clinical presentation, medical history, and vaccination history were systematically obtained from parents or guardians using a structured proforma specifically designed for this study by the pediatric research team at the Department of Pediatrics, Mardan Medical Complex (Table 1). The proforma captured detailed information on socio-demographics, clinical features, laboratory results, treatment, and outcomes. It was pilot-tested on 10 cases to assess clarity, feasibility, and completeness, and minor adjustments were made to optimize usability, although it was not formally validated.

**Table 1:** Structured questionnaire/proforma for children with suspected bacterial meningitis. Hib: Haemophilus influenzae type b; PCR: polymerase chain reaction; EPI: Expanded Program on Immunization; BCG: bacillus Calmette-Guérin; DTP: diphtheria, tetanus, and pertussis; CSF: cerebrospinal fluid.

Section	Variable	Details/response options	Notes
Demographics	Patient ID		
	Age (months)	___	
	Sex	Male/Female	
	Weight (kg)	___	
	Height (cm)	___	
	Residence	Urban/Rural	
	Socioeconomic status	Low/Middle/High	Based on family income/occupation
Clinical presentation	Date of admission	___	DD/MM/YYYY
	Fever	Yes/No	
	Neck stiffness	Yes/No	
	Altered consciousness	Yes/No	
	Seizures	Yes/No	
	Vomiting	Yes/No	
	Other symptoms	___	
Vaccination history	Hib vaccine	Fully/Partially/Not vaccinated	Verified by EPI card or parental recall
	PCV10 (pneumococcal)	Fully/Partially/Not vaccinated	Specify dates if available
	Neisseria meningitidis vaccine	Not routine in Pakistan	Optional
	Other vaccines	___	e.g., BCG and DTP
	Vaccination card available	Yes/No	
Medical history	Prior antibiotic use	Yes/No	If yes, specify drug & duration
	Chronic illness	Yes/No	If yes, specify
	Recent hospitalization	Yes/No	Specify duration
Laboratory findings	CSF collection date	___	DD/MM/YYYY
	CSF cytology	WBC count/Differential	
	CSF biochemistry	Protein, Glucose	
	CSF culture result	Organism name/No growth	
	Gram stain	Positive/Negative	Specify organism if positive
	PCR for bacterial pathogens	Not available/NA	Optional if future use
Outcome/follow-up	Diagnosis confirmed	Yes/No	Based on CSF results
	Treatment given	___	
	Outcome	Recovered/Complications/Death	

Vaccination verification and categorization

Vaccination status was verified primarily via immunization cards. When cards were unavailable, parental recall was accepted but carefully documented as a potential source of bias. Only consistent and verifiable details, such as vaccine type, date, and number of doses, were included under parental recall, while uncertain responses were excluded as unverifiable. Children were categorized as vaccinated (received at least the Hib and PCV10 vaccines according to the national EPI schedule), partially vaccinated (received only one of these vaccines), and unvaccinated (received neither). *Neisseria meningitidis* vaccination is not routinely part of Pakistan’s EPI schedule and was not used for categorization.

Contextual considerations in Pakistan

In routine practice, vaccines in Pakistan are sometimes administered by non-health professionals, such as teachers, clerks, or newly recruited staff, who may lack sufficient training. Improper storage, handling, or administration in such settings can reduce vaccine effectiveness and contribute to residual cases of vaccine-preventable diseases. This emphasizes the need for strengthening healthcare training, supervision, and cold chain management to ensure optimal vaccine impact.

Additional measures

All data were cross-checked daily for completeness and consistency by the study team. Any discrepancies or missing entries were clarified with the attending clinician or guardian before final entry. Confidentiality was maintained by assigning unique identifiers to each child, and all hardcopy and electronic records were stored securely.

Statistical analysis

Data were entered and analyzed using SPSS version 26 (IBM Corp., Armonk, NY). Descriptive statistics included frequencies and percentages for categorical variables and means with standard deviations (SDs) for continuous variables, and 95% confidence intervals (CIs) were calculated for mean ages to assess group comparability. Baseline differences in age and sex between vaccinated and unvaccinated groups were formally tested using independent t-tests and chi-square tests, respectively.

The association between vaccination status and confirmed bacterial meningitis was quantified using odds ratios (ORs) with 95% CIs, and group comparisons were assessed with the chi-square test. Given the cross-sectional design, ORs were interpreted as approximations of prevalence ratios, rather than direct measures of risk. Statistical significance was defined as p < 0.05.

While the ORs presented are unadjusted, future studies should consider adjusting for potential confounders, such as age, nutritional status, socioeconomic background, prior antibiotic use, and comorbidities, to improve causal inference. Multiple testing corrections were not applied to pathogen-specific subgroup analyses; therefore, these results should be interpreted with caution.

Missing data were minimal (<5%) and were addressed using case-wise exclusion. Sensitivity analyses were not performed due to the low frequency of missing values, reducing the potential for bias. All steps were designed to ensure transparency, reproducibility, and reliable interpretation of the findings.

Ethical approval

The study protocol was reviewed and approved by the Institutional Review Board (IRB)/Ethics Committee, Mardan Medical Complex, Mardan (Approval No.: 807/ORT/BKMC; Date: 21.12.2022). Written informed consent was obtained from parents or guardians of all participants. Confidentiality of patient information was ensured, and all data were anonymized prior to analysis.

## Results

Out of 280 enrolled children, 168 (60.00%) were vaccinated and 112 (40.00%) were unvaccinated (Table 2). The mean age of vaccinated children was 28.35 ± 12.17 months (95% CI: 26.51-30.19), while that of unvaccinated children was 30.06 ± 12.62 months (95% CI: 27.72-32.40). The difference in mean ages was not statistically significant (independent t-test, p = 0.27). Among vaccinated children, 97/168 (57.74%) were males and 71/168 (42.26%) were females, whereas in the unvaccinated group, 67/112 (59.82%) were males and 45/112 (40.18%) were females. The gender distribution between groups was also not significantly different (chi-square test, p = 0.72), confirming comparability of baseline characteristics.

**Table 2:** Baseline characteristics of the study population (n = 280). Independent t-test was applied for age, and Pearson’s chi-square test was used for gender distribution. * Statistically significant at p < 0.05.

Variable	Vaccinated (n = 168)	Unvaccinated (n = 112)	Test statistic	p-value
Age (months), Mean ± SD (95% CI)	28.35 ± 12.17 (26.51–30.19)	30.06 ± 12.62 (27.72–32.40)	t(278) = –1.10	0.27
Gender, Male, n (%)	97 (57.7%)	67 (59.8%)	χ²(1) = 0.13	0.72
Gender, Female, n (%)	71 (42.3%)	45 (40.2%)	–	–
Total	168 (100%)	112 (100%)	–	–

**Figure 1:**
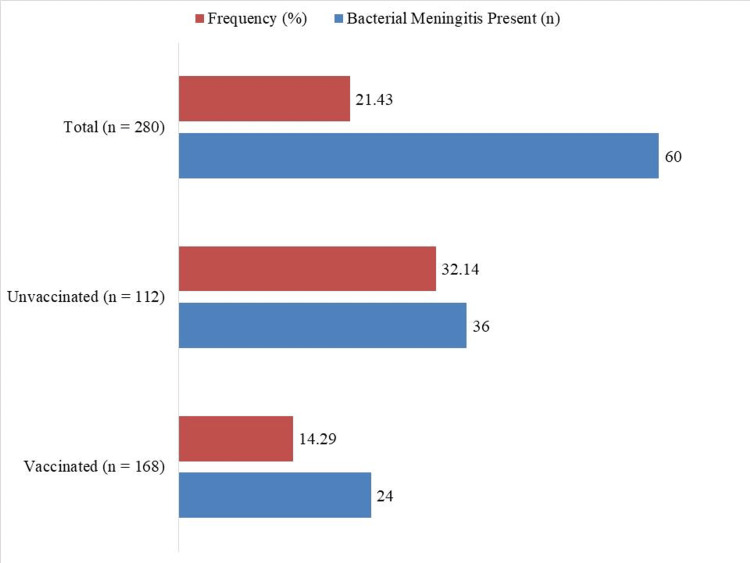
Frequency of confirmed bacterial meningitis by vaccination status. Bacterial meningitis was confirmed in 60/280 (21.43%) children. Frequency was significantly lower among vaccinated children (14.29%) compared to unvaccinated children (32.14%) (χ²(1) = 11.87, p = 0.0006).

Bacterial meningitis was confirmed in 60 (21.43%) out of 280 children (Figure 1). The frequency was significantly lower among vaccinated children, 24 out of 168 (14.29%), compared to unvaccinated children, 36 out of 112 (32.14%), highlighting the protective effect of vaccination against bacterial meningitis.

Among the 60 confirmed cases, *Streptococcus pneumoniae* was the predominant pathogen, isolated in 24/60 (40.0%) children, including 11/24 (45.83%) in the vaccinated group and 15/36 (41.67%) in the unvaccinated group (Table 3). Hib was detected in 13/60 (21.67%) cases, including 4/24 (16.67%) vaccinated and 9/36 (25.00%) unvaccinated children. *Neisseria meningitidis* was found in 8/60 (13.33%) cases, more frequent in unvaccinated (6/36; 16.67%) than vaccinated children (2/24; 8.33%). Gram-negative organisms included *Escherichia coli* in 7/60 (11.67%), *Klebsiella pneumoniae* in 4/60 (6.67%), and other bacilli in 2/60 (3.33%). Differences in pathogen distribution between vaccinated and unvaccinated groups were not statistically significant (Fisher’s exact test, p = 0.41).

**Table 3:** Distribution of causative organisms identified in cerebrospinal fluid cultures (n = 60).

Organism	Vaccinated (n = 24)	Unvaccinated (n = 36)	Total (n = 60)
Streptococcus pneumoniae	11 (45.8%)	15 (41.7%)	26 (43.3%)
Haemophilus influenzae type b	4 (16.7%)	9 (25.0%)	13 (21.7%)
Neisseria meningitidis	2 (8.3%)	6 (16.7%)	8 (13.3%)
Escherichia coli	4 (16.7%)	3 (8.3%)	7 (11.7%)
Klebsiella pneumoniae	2 (8.3%)	2 (5.6%)	4 (6.7%)
Other Gram-negative bacilli	1 (4.2%)	1 (2.8%)	2 (3.3%)
Total	24 (100%)	36 (100%)	60 (100%)

Comparison of vaccination status with bacterial meningitis occurrence showed that unvaccinated children were significantly more likely to develop meningitis, with 36/112 (32.14%) cases compared to 24/168 (14.29%) in the vaccinated group (Table 4). The calculated odds ratio was 2.85 (95% CI: 1.56-5.21), with χ² = 11.87 and p = 0.0006. This odds ratio is unadjusted and reflects an approximation of the prevalence ratio in this cross-sectional study. Adjustment for potential confounders (age, nutrition, socioeconomic status) was not performed, but is recommended for future studies.

**Table 4:** Association between vaccination status and bacterial meningitis. Pearson’s chi-square test was used to assess differences between groups, and the odds ratio (OR) reported is unadjusted, serving as an approximation of the prevalence ratio in this cross-sectional study (*p < 0.05, statistically significant). Outcomes among children with bacterial meningitis were categorized as follows: recovered, for those who fully recovered without sequelae; complications, for children who developed neurological or systemic sequelae such as hearing loss, cognitive impairment, seizures, motor deficits, or other clinically documented adverse events; and death, for children who died during hospitalization due to bacterial meningitis or its immediate complications.

Vaccination status	Bacterial meningitis present, n (%)	Bacterial meningitis absent, n (%)	Total	OR (95% CI)	χ²	p-value	Outcome: Recovered, n (%)	Outcome: Complications, n (%)	Outcome: Death, n (%)
Vaccinated (n = 168)	24 (14.3%)	144 (85.7%)	168	2.85 (1.56–5.21)	11.87	0.0006*	18 (75.0%)	4 (16.7%)	2 (8.3%)
Unvaccinated (n = 112)	36 (32.1%)	76 (67.9%)	112	–	–	–	25 (69.4%)	7 (19.4%)	4 (11.1%)
Total	60 (21.4%)	220 (78.6%)	280	–	–	–	43 (71.7%)	11 (18.3%)	6 (10.0%)

## Discussion

Our study's results demonstrate the important role that immunization plays in lowering the prevalence of bacterial meningitis in children. There was a noticeable difference between the vaccinated and unvaccinated groups in our cohort of 280 children, with 60 (21.43%) receiving a confirmed diagnosis of bacterial meningitis. With an OR of 2.85 (95% CI: 1.56-5.21) and a p-value of 0.0006, the incidence was significantly lower in vaccinated children (14.29%) than in unvaccinated children (32.14%), indicating a statistically significant protective effect of vaccination. Although ORs in cross-sectional studies reflect associations rather than causality, they provide a useful approximation of prevalence ratios in this context. The importance of routine vaccination regimens in preventing major bacterial illnesses is further supported by these findings. Similar results have been reported elsewhere; for instance, Scelfo et al. [13] found that pneumococcal conjugate vaccination (PCV) significantly reduced the incidence of meningitis caused by *Streptococcus pneumoniae*, demonstrating that immunization programs considerably lower the burden of illness, even in resource-limited settings.

The most common causative organism in our study was *Streptococcus pneumoniae*, identified in 24 children (11 vaccinated and 15 unvaccinated). Hib was found in 13 cases (four vaccinated and nine unvaccinated), and *Neisseria meningitidis* was found in eight cases (two vaccinated and six unvaccinated). Despite extensive immunization, *Streptococcus pneumoniae* remains a leading cause of pediatric bacterial meningitis, consistent with global epidemiological trends [14,15]. The higher incidence of Hib and *Neisseria meningitidis* in unvaccinated children highlights the importance of vaccination, particularly for pathogens targeted by Pakistan’s EPI. Gram-negative bacteria, including *Klebsiella pneumoniae* and *Escherichia coli*, were less prevalent, aligning with reports that these organisms are more commonly associated with neonatal or immunocompromised populations [16].

Our study's demographic distribution showed that the mean age of vaccinated children was 28.35 ± 12.17 months, while that of unvaccinated children was 30.06 ± 12.62 months. Both groups were male-dominated (57.74% and 59.82%, respectively), consistent with previous studies of pediatric bacterial meningitis [12]. The similarity in baseline characteristics strengthens the validity of our findings on the protective impact of vaccination.

Challenges in achieving full vaccination coverage were also evident. Despite a lower incidence of meningitis, 14.29% of vaccinated children still developed the illness, likely due to incomplete vaccination schedules, vaccine failure, or circulation of non-vaccine serotypes. This is corroborated by earlier research that showed breakthrough pneumococcal meningitis in children who got the whole vaccination regimen, but at lower rates than in those who were not immunized [17]. The increased incidence of bacterial meningitis in children who have not received vaccinations highlights the pressing need to address vaccination-related obstacles such as vaccine hesitancy, ignorance, and logistical difficulties in rural regions.

Additionally, in Pakistan, vaccines are frequently administered by non-health professionals such as teachers, clerks, or newly trained personnel who may lack adequate knowledge and training, leading to mishandling or improper storage of vaccines. This factor may contribute to reduced vaccine effectiveness and emphasizes the need for strengthened training and monitoring within immunization programs. The higher incidence of bacterial meningitis among unvaccinated children underscores the urgent need to address barriers to vaccination, including vaccine hesitancy, lack of awareness, logistical challenges, and limited access in rural areas.

Multiple testing corrections were not applied to pathogen-specific subgroup analyses; hence, these results should be interpreted with caution. Future studies with larger sample sizes should adjust for multiple comparisons and potential confounders such as age, nutrition, and socioeconomic status. Our findings provide empirical evidence that vaccination significantly reduces a child’s risk of bacterial meningitis. They also highlight the importance of maintaining high vaccination coverage and implementing focused public health interventions, including improved training for vaccine providers, outreach programs to unvaccinated communities, and continuous monitoring of immunization quality. Future research should also consider serotype determination, longitudinal assessment of vaccine effectiveness, and strategies to increase vaccine uptake.

Study strengths and limitations

This study draws strength from a well-defined single-center cohort of 280 children with clearly verified vaccination status, allowing for reliable comparisons between vaccinated, partially vaccinated, and unvaccinated groups. Diagnostic confirmation using cerebrospinal fluid analysis, including cytology, biochemistry, and culture, enhances the validity of findings and reduces misclassification risk. Consecutive sampling minimized selection bias, and comparable demographic characteristics between groups strengthened the robustness of comparisons.

However, several limitations should be noted. The single-center design may limit generalizability to other regions of Pakistan with differing healthcare access and vaccination coverage. Reliance on parental recall for vaccination history in some cases introduces potential recall bias, although inconsistent responses were excluded to partially mitigate misclassification. Serotype-specific analysis of bacterial isolates was not performed; future studies, including serotype determination, are warranted to better understand vaccine effectiveness, epidemiological trends, and potential emergence of non-vaccine serotypes. Prior antibiotic use before admission may have reduced culture yield, and seasonal variation could have influenced observed prevalence. Finally, multiple comparisons in pathogen-specific analyses were not adjusted statistically, which may increase the risk of type I error.

## Conclusions

The study highlights the significant protective effect of vaccination against bacterial meningitis in young children. Vaccinated children were considerably less likely to develop the disease, emphasizing the effectiveness of routine immunization programs. *Streptococcus pneumoniae* remained the most common causative organism, followed by Hib and *Neisseria meningitidis*, reinforcing the importance of vaccines targeting these pathogens. Despite these findings, residual cases among vaccinated children underline the need for continued surveillance, evaluation of vaccine effectiveness against non-vaccine serotypes, and strategies to improve timely completion of immunization schedules. Seasonal variation, recall bias, and prior antibiotic use should also be considered in interpreting hospital-based data. These findings underscore the need to enhance immunization coverage, address barriers to vaccine access, and strengthen public health strategies to reduce the burden of preventable bacterial meningitis in children.
